# Dynamic causal modelling of lateral interactions in the visual cortex

**DOI:** 10.1016/j.neuroimage.2012.10.078

**Published:** 2013-02-01

**Authors:** D.A. Pinotsis, D.S. Schwarzkopf, V. Litvak, G. Rees, G. Barnes, K.J. Friston

**Affiliations:** The Wellcome Trust Centre for Neuroimaging, University College London, Queen Square, London WC1N 3BG, UK

**Keywords:** Neural field theory, Dynamic causal modelling, MEG, Connectivity, Visual cortex, Electrophysiology

## Abstract

This paper presents a dynamic causal model based upon neural field models of the Amari type. We consider the application of these models to non-invasive data, with a special focus on the mapping from source activity on the cortical surface to a single channel. We introduce a neural field model based upon the canonical microcircuit (CMC), in which neuronal populations are assigned to different cortical layers. We show that DCM can disambiguate between alternative (neural mass and field) models of cortical activity. However, unlike neural mass models, DCM with neural fields can address questions about neuronal microcircuitry and lateral interactions. This is because they are equipped with interlaminar connections and horizontal intra-laminar connections that are patchy in nature. These horizontal or lateral connections can be regarded as connecting macrocolumns with similar feature selectivity. Crucially, the spatial parameters governing horizontal connectivity determine the separation (width) of cortical macrocolumns. Thus we can estimate the width of macro columns, using non-invasive electromagnetic signals. We illustrate this estimation using dynamic causal models of steady-state or ongoing spectral activity measured using magnetoencephalography (MEG) in human visual cortex. Specifically, we revisit the hypothesis that the size of a macrocolumn is a key determinant of neuronal dynamics, particularly the peak gamma frequency. We are able to show a correlation, over subjects, between columnar size and peak gamma frequency — that fits comfortably with established correlations between peak gamma frequency and the size of visual cortex defined retinotopically. We also considered cortical excitability and assessed its relative influence on observed gamma activity. This example highlights the potential utility of dynamic causal modelling and neural fields in providing quantitative characterisations of spatially extended dynamics on the cortical surface — that are parameterised in terms of horizontal connections, implicit in the cortical micro-architecture and its synaptic parameters.

## Highlights

► New neural field model of canonical cortical microcircuitry ► Modelling of vertical interlaminar and horizontal intralaminar connections ► Estimation of the width of macrocolumns using non-invasive (MEG) signals ► Assessment of influence of cortical excitability and microcircuitry to gamma peak ► Mechanistic approach to correlations among cortical structure and function.

## Introduction

This work combines neural field models — that model the activity of layers of cells in cortical patches — with a Bayesian framework for optimising model parameters — known as Dynamic Causal Modelling (DCM). This combination allows one to address questions about lateral cortical interactions in terms of optimal models and model parameters. DCM has been applied extensively to fMRI and electrophysiological data (David et al., 2006; Friston et al., 2003; Penny et al., 2004) and has been used recently to model spatiotemporal dynamics on the cortical surface (Pinotsis et al., 2012). Combining generative models with Bayesian optimisation techniques enables one to characterise the functional architectures that generate empirical data. In this paper, we show that DCM can disambiguate between alternative spatiotemporal models of cortical activity — using Bayesian model comparison — and furnish quantitative explanations of observed responses, in terms of the biophysical properties of lateral cortical connections. This work focuses on two classes of biophysical models describing mesoscale brain activity: neural mass and field models. Neural field models describe how hidden neuronal states (such as the average depolarisation of a neural population or layer) evolve over both space and time. In contrast, neural mass models only characterise dynamics over time, under the assumption that all the neurons of a particular population are located at (approximately) the same point.

In previous work (Pinotsis et al., 2012), we considered the relationship between neural mass and field models and showed that field models can be reduced to neural masses by applying shrinkage priors to spike propagation times — such that lateral interactions were effectively instantaneous. We introduced a DCM that provides an explicit model of spatially extended cortical activity that allows one to make inferences about key parameters controlling the topographic distribution of cortical activity using LFP data, like the extent of lateral cortical connections and the conduction velocity of spike propagation. We were able to show that including spatial parameters enables one to explain effects that other models — such as neural mass models — attribute to variations in temporal parameters, like synaptic rate constants. Dynamic causal models based on neural fields enable one to characterise the propagation of activity on the cortex and provide a formal understanding of the mechanisms generating spatiotemporal responses. In our previous work above, we considered model optimisation under the assumption that one could measure local signals (local field potentials) that are sensitive to all spatial frequencies. In this paper, we consider neural field models of non-invasive (EEG or MEG) data. This development allows one to make inferences about the nature of lateral interactions in cortical sources, without spatially resolved measurements.

### Spectral responses of neural fields

The characterisation of electrophysiological signals depends upon models of how they are generated in source space and how the resulting (hidden) neuronal states are detected by sensors. At the source level we consider a model based upon a canonical microcircuit that allows one to separate the sources of forward and backward connections in cortical hierarchies. In terms of the mapping from source to sensor space — we use a conventional lead field formulation that is expanded in terms of spatial basis functions. As in previous work, we focus on the modelling of power spectra. There is a long history of modelling steady-state (or ongoing) activity spectra, associated with neural fields, usually in models of the whole cortex, e.g., (Jirsa, 2009). Robinson (2006) has developed a neurophysiologically grounded model of corticothalamic activity, which reproduces many properties of empirical EEG signals; such as the spectral peaks seen in various sleep states and seizure activity. Technically, the spectra summarising the response of cortical sources can be defined in terms of transfer functions, mapping endogenous neuronal fluctuations to observed responses (Freeman, 1972; Nunez, 1995; Robinson et al., 2001; Robinson et al., 2003). In Pinotsis and Friston (2011), we derived the transfer function — and an expression for the spectral responses — for a source described by a classical neural field equation, while in Pinotsis et al. (2012) we extended our approach to a cortical source that comprises multiple layers. This allowed us to model the spectral activity of cortical fields as measured on the cortical surface. Here, we pursue a similar approach and generate the corresponding spectral responses, as measured by non-invasive sensors. These predictions are generated in an efficient manner that exploits the nature of the mapping from sources to sensors in EEG and MEG.

The resulting scheme can be regarded as inverting or fitting population models of the Amari type, using real data and Bayesian model inversion. Previous work in a similar vein includes the use of Kalman filters to develop estimation schemes for both neural mass (Riera et al., 2007; Valdes et al., 1999) and neural field models of a single population (Galka et al., 2008; Schiff and Sauer, 2008). In a related approach, Daunizeau et al. (2009)) replaced the standard dipole source — used in neural mass models — with the principal Fourier mode of a neural field, for the particular case of exponentially decaying synaptic density over the cortical surface. Finally (Markounikau et al. (2010) used a combination of linear and nonlinear optimisation methods to invert a two-layered neural field model of voltage-sensitive dye data, describing inhibitory and excitatory populations (without conduction delays). The neural field model considered here has four layers and is based on canonical cortical microcircuitry that accounts for several aspects of local cortical computations in theoretical neurobiology. This model provides an extension of the well known Jansen and Rit neural mass model and incorporates conduction delays associated with the propagation of neuronal spikes.

### Lateral interactions and neural fields

Modelling lateral interactions with neural field models has a long history. Pioneering work was introduced in papers by Amari, Wilson and Cowan, Grossberg and colleagues (Amari and Arbib, 1977; Amari and Takeuchi, 1978; Grossberg and Levine, 1975; Wilson and Cowan, 1973). These developments can be traced back to the work of physicists in the 19th century — such as Helmholtz and Mach — on visual perception. The first neural field models considered spontaneous pattern formation, by analysing the steady-state behaviour of underlying field equations: for example, Wilson and Cowan developed a treatment of Turing instabilities in the context of neural fields (Wilson and Cowan, 1972). In a similar vein, Grossberg initiated a line of work on shunting interactions — via nonlinearly coupled inputs — and considered limits as the solutions approached steady state (Grossberg, 1973). At about the same time, Amari proved that systems of neural fields typically approach steady-state, in which some parts remain active, thus providing a metaphor for short-term memory.

From an anatomical viewpoint, the functional specialisation of visual (and auditory) cortex is reflected in its patchy or modular organisation — in which local cortical structures share common response properties. This organisation may be mediated by a patchy distribution of horizontal intrinsic connections that can extend up to 8 mm, linking neurons with similar receptive fields: see, e.g., Angelucci and Bressloff (2006), Burkhalter and Bernardo, (1989), and Wallace and Bajwa (1991). The existence of patchy connections in different cortical areas (and species) has been established with tracer studies in man, macaque and cat: Burkhalter and Bernardo (1989), Stettler et al. (2002) and (Wallace and Bajwa (1991), respectively. It has been shown that such connections can have profound implications for neural field dynamics: see Baker and Cowan (2009). The precise form of such connections may be explained by self-organisation under functional and structural constraints; for example, minimising the length of myelinated axons to offset the cost of transmitting action potentials (Cherniak, 1994; Wen and Chklovskii, 2008). Generic constraints of this sort have been used to motivate general principles of connectivity; namely, that evolution attempts to optimise a trade-off between metabolic cost and topological complexity (Bassett et al., 2010). In short, visual and auditory cortices can be characterised by a patchy organisation that is conserved over the cortex and which allows for both convergence and divergence of cortical connections. Synaptic densities can then be approximated by isotropic distributions with an exponential decay over the cortical surface. In this work, we use a combination of patchy and isotropic distributions, using connectivity kernels with non-central peaks to model sparse intrinsic connections in cortical circuits that mediate both local and non-local interactions. In other words, we consider models in which neurons receive signals both from their immediate neighbours and remote populations that share the same functional selectivity (Pinotsis and Friston, 2011). We focus on the particular problem of identifying the parameters of lateral connections within a bounded cortical patch or manifold, as measured at a distance, with an array of non-invasive (MEG or EEG) sensors.

This paper comprises three sections. The first reviews a canonical neural mass model based on anatomical data and theoretical constraints from the theory of predictive coding. The second section describes a neural field model based upon this canonical microcircuitry. Our focus here is on equipping the resulting neuronal model with an electromagnetic forward model to predict responses in non-invasive sensors. In the third section, we use Bayesian model comparison to adjudicate between various formulations of the ensuing neural field model (DCM) and establish its construct validity by optimising parameters pertaining to GABAergic concentrations, that have been shown to correlate with the peak gamma frequency of steady-state activity (Muthukumaraswamy et al., 2009).

## A canonical model of cortical activity

This section introduces the neuronal field model used in subsequent sections on dynamic causal modelling. The particular neuronal model of source activity used here is based upon a refinement of conventional (convolution–based) neuronal models that explicitly model the neuronal sources of forward and backward connections in cortical hierarchies — these are the superficial and deep pyramidal cell populations respectively. We develop the corresponding neural field model from established neural mass models.

### The canonical microcircuit

Recent work suggests that superficial layers of visual cortex oscillate at gamma frequencies, while deep layers primarily oscillate at lower frequencies (Buffalo et al., 2011). Since forward connections originate predominately from superficial layers and backward connections primarily originate in deep layers, these spectral asymmetries suggest that forward connections use faster (gamma) temporal frequencies, while backward connections may employ lower (alpha or beta) frequencies — a suggestion that has experimental support (Roopun et al., 2010). These asymmetries mandate something quite remarkable: namely, a synthesis and segregation of forward and backward efferents from afferent input. This segregation can only arise from local non-linear neuronal computations that are structured and precisely interconnected. Non-linear neuronal transformations are necessary to account for the implicit coupling between different frequencies and arise from both synaptic mechanisms (e.g. non-linear dendritic integration) and population dynamics (e.g. sigmoid activation functions). The canonical microcircuit is a detailed proposal for such a laminar-specific intracortical architecture that describes how information flows through the cortical column. This model is based on findings in the primary visual cortex (Douglas and Martin, 1991) but recent work (Lefort et al., 2009; Weiler et al., 2008) indicates that similar microcircuits exist in other regions, such as somatosensory and motor cortex.

Douglas and Martin recorded intracellular potentials from cells in area 17 of the cat while they stimulated cortical afferents and noticed a strong compartmentalisation of the superficial and deep cell properties — reflected in slow superficial responses and fast input layer responses. The authors created a conductance-based model that reproduced the evolution of excitation and inhibition through the cortical circuit with great precision. This model contained three groups of cells: superficial and deep pyramidal cells, and a common pool of inhibitory cells. All three pools of neurons receive thalamic drive — although the thalamic drive to deep layer cells was weaker than the others. All neuronal populations expressed significant self-connectivity, and interconnected with the other populations. This model reproduced the features observed in their electrophysiological recordings — including the latency difference between superficial and deep layer neurons — and has served to establish several basic properties that are now believed to be replicated in other cortical areas: first, although superficial and deep compartments are strongly interconnected, their response properties are also strongly segregated. Second, cortex is not under tonic inhibition, rather, both excitation and inhibition are generated by afferent thalamic input and both shape ongoing cortical responses. Third, the canonical microcircuit can amplify thalamic inputs to generate self-sustaining activity, while also maintaining a delicate balance between excitation and inhibition — so as to prevent runaway excitation.

Previous computational modelling studies indicate that this circuitry allows the cortex to optimally organise and integrate bottom-up, lateral, and top-down information (Raizada and Grossberg, 2003). Douglas and Martin suggest that the rich anatomical connectivity of superficial layer (2/3) pyramidal cells allows them to collect information from all relevant top-down, lateral, and bottom-up inputs, and — through processing in the dendritic tree — select the most likely interpretation of its subcortical inputs. For a relevant discussion and more details on the canonical microcircuit and its potential role in predictive coding we refer the reader to Bastos et al. (under review).

**Fig. 1:**
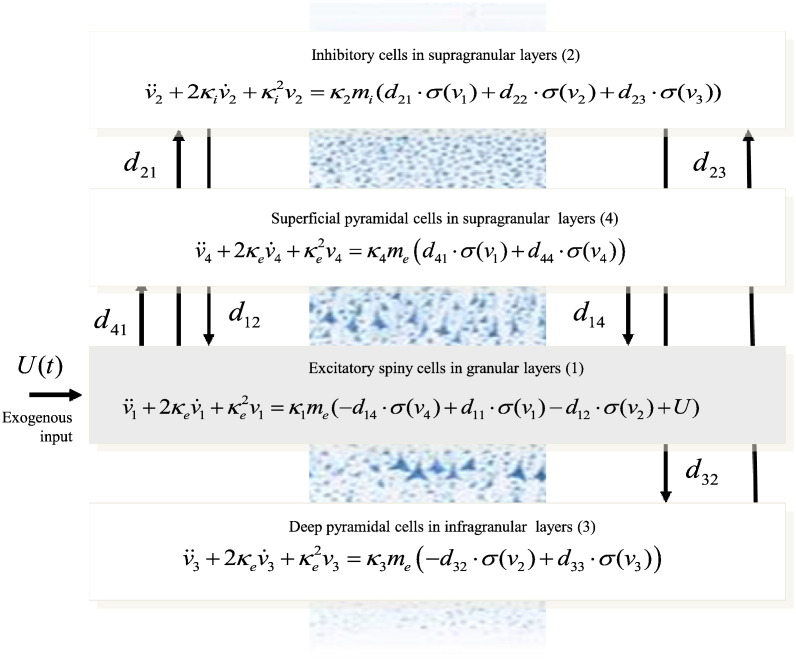
The Canonical Microcircuit (CMC) neural mass model. This figure shows the evolution equations that specify a CMC mass model of a single source. This model contains four populations occupying different cortical layers: the pyramidal cell population of the JR model is here split into two subpopulations allowing a separation of the sources of forward and backward connections in cortical hierarchies. As with the JR model, second-order differential equations mediate a linear convolution of presynaptic activity to produce postsynaptic depolarisation. This depolarisation gives rise to firing rates within each sub-population that provide inputs to other populations.

Haeusler and Maass used Hodgkin and Huxley neurons to build a realistic microcircuit model and showed that a cortical column — whose connectivity conforms to the canonical microcircuit — can perform various computations efficiently, in relation to a column with random connectivity (Haeusler and Maass, 2007). By collapsing two pairs of cell types in the Haeusler and Maass model — while preserving the topology of the connectivity — one obtains the canonical microcircuit depicted in Fig. 1: this circuit comprises four populations: excitatory spiny stellate input cells (1), inhibitory interneurons (2), deep excitatory output pyramidal cells (3) and superficial excitatory pyramidal cells (4). In what follows, we describe a mathematical model of how the neuronal states of this these populations evolve over time. This model provides the basis of our dynamic causal model:We first define the vector-valued function

(1) $$V={\left(v_{1},v_{2},v_{3},v_{4}\right)}^{T}$$

where *v*_*a*_(*t*) denotes the expected depolarisation of the *a*-th population (*a* = 1,…,4) at time *t*. The dynamics of depolarisation are then described by the following second-order differential equation:

(2) $$\ddot{V}+2B\dot{V}=-B^{2}V+AB\cdot F\circ V+GU$$

where $U\left(t\right)\in R^{4}$ is a vector of external inputs (exogenous neuronal fluctuations) and *A* and *B* are 4 × 4 matrices of synaptic parameters controlling the maximum postsynaptic responses for excitatory (*m*_*e*_) and inhibitory (*m*_*i*_) populations and the rate-constants of postsynaptic filtering (*κ*_1_, …, *κ*_4_; c.f., decay):

(3) $$\begin{array}{c} A=diag\left(m_{e},m_{i},m_{e},m_{e}\right)\\ B=diag\left(\kappa_{1},\kappa_{2},\kappa_{3},\kappa_{4}\right) \end{array}$$

$F:R^{4}\to R^{4}$ is a nonlinear mapping from postsynaptic depolarisation to presynaptic firing rates and $G:R^{4}\to R^{4}$ maps the inputs to the motion of hidden neuronal states; namely

(4) $$G={\left(\kappa_{1}m_{e},0,0,0\right)}^{T}$$

In summary, Eq. (2) expresses the rate of change of expected depolarisation in each population as a sum of three terms; the first is a simple decay, the second is due to presynaptic input from other parts of the cortex and the final part is due to external inputs *U*(*t*). Writing out Eq. (2) in component form, we have

(5) $$\begin{array}{c} {\ddot{v}}_{1}+2\kappa_{e}{\dot{v}}_{1}+\kappa_{e}^{2}v_{1}=\kappa_{1}m_{e}\left(-d_{14}\cdot \sigma \left(v_{4}\right)+d_{11}\cdot \sigma \left(v_{1}\right)-d_{12}\cdot \sigma \left(v_{2}\right)+U\right){\ddot{v}}_{2}+2\kappa_{i}{\dot{v}}_{2}+\kappa_{i}^{2}v_{2}=\kappa_{2}m_{i}\left(d_{21}\cdot \sigma \left(v_{1}\right)+d_{22}\cdot \sigma \left(v_{2}\right)+d_{23}\cdot \sigma \left(v_{3}\right)\right){\ddot{v}}_{3}+2\kappa_{e}{\dot{v}}_{3}+\kappa_{e}^{2}v_{3}=\kappa_{3}m_{e}\left(-d_{32}\cdot \sigma \left(v_{2}\right)+d_{33}\cdot \sigma \left(v_{3}\right)\right){\ddot{v}}_{4}+2\kappa_{e}{\dot{v}}_{4}+\kappa_{e}^{2}v_{4}=\kappa_{4}m_{e}\left(d_{41}\cdot \sigma \left(v_{1}\right)+d_{44}\cdot \sigma \left(v_{4}\right)\right) \end{array}$$

where the sigmoid firing rate function is

(6) $$\sigma \left(v_{a}\right)=\frac{1}{1+\exp\left(r\left(\eta -v_{a}\right)\right)}$$

Here, *r* and *η* are parameters that determine the shape of this sigmoid activation function. In particular, *r* is synaptic gain and *η* is the postsynaptic potential at which the half of the maximum firing rate is elicited. In Eq. (5), *d*_*ab*_ ⋅ *σ*(*v*_*b*_) is (endogenous) presynaptic input to the *a*-th population from the *b*-th and corresponds to the mapping *F* ∘ *V*. This is a sigmoid function *σ*(*v*_*b*_) of postsynaptic depolarisation in the *b*-th population, multiplied by the intrinsic connection strength *d*_*ab*_ between the two populations (Jansen and Rit, 1995). See Fig. 1 for a schematic of this model.

### A canonical microcircuit field model and its transfer function

In the case of neural field models, we consider spatially extended sources occupying bounded manifolds (patches) in different layers that lie beneath the cortical surface. In this setting, each population now becomes a layer in the cortical sheet. Here, the input *U*(*x*,*t*) is an explicit function of both space and time. Furthermore, the 4 × 1 vector $V\left(t\right)\in R^{4}$ pertaining to the hidden neuronal states of each layer is replaced by $V\left(x,t\right)\in R^{4}$; a vector field depending on both time and space. The dynamics of cortical sources now conform to integrodifferential equations, such as the Wilson-Cowan or Amari equations, where coupling is parameterised by matrix-valued coupling kernels — namely, smooth (analytic) connectivity matrices that also depend on time and space. These spatial generalisations can be summarised in the following equation, which is the analogue of Eq. (2) for neural field models:

(7) $$\ddot{V}=-2B\dot{V}-B^{2}V+AB\iint D\left(x-x^{\prime },t-t^{\prime }\right)\cdot F\circ V\left(x^{\prime },t^{\prime }\right)dx^{\prime }dt^{\prime }+G\circ U$$

where the elements of the connectivity matrix *D*(*x*,*t*) are *d*_*ab*_(*x*,*t*) = *k*_*ab*_(*x*)*δ*(*t* − |*x*|*υ*). This spatiotemporal matrix corresponds to the first-order approximation to the composition of the spatial kernels *K*(*x*) defined below and the temporarily delayed firing rates. Eq. (7) can be written as

(7’) $$\left(\ddot{V}+2B\dot{V}+B^{2}V\right)\left(x,t\right)=AB\int K\left(x-x'\right)F\circ V\left(x',t-|x-x'|\upsilon \right)dx'+G\circ U$$

where *υ* is the inverse speed with which spikes propagate along connections and interactions among populations — within and across macrocolumns — are described by the connectivity kernel *K* = *K*^(*i*)^ + *K*^(*e*)^. One can see that in the infinite speed limit *υ* = 0 the spatial convolution in the above Equation disappears (to within a scaling constant) and we recover the neural mass eq. (5). In this limit, the corresponding electrophysiological predictions effectively coincide with those generated by a neural mass model. We will use prior constraints on the propagation speed below to compare field and mass variants of the canonical microcircuit (for a fuller discussion, see Pinotsis et al., 2012). The intrinsic part *K*^(*i*)^ is an exponentially decaying kernel commonly used in the literature to account for excitatory and inhibitory interactions (see e.g. Pinotsis et al., 2012), while the extrinsic part of the kernel *K*^(*e*)^ was introduced in Grindrod and Pinotsis, (2011) and Pinotsis and Friston (2011) to model patchy lateral connections. This kernel is characterised by non-central peaks allowing for differences in (and estimation of) the range and dispersion of lateral connections, summarised in terms of the parameters *h*_*a*_ and *c*_*aa*_ respectively, namely

(8) $$\begin{array}{c} K^{\left(i\right)}=\left[\begin{array}{cccc} k_{11}^{\left(i\right)} & k_{12}^{\left(i\right)} & 0 & k_{14}^{\left(i\right)}\\ k_{21}^{\left(i\right)} & k_{22}^{\left(i\right)} & k_{23}^{\left(i\right)} & 0\\ 0 & k_{32}^{\left(i\right)} & k_{33}^{\left(i\right)} & 0\\ k_{41}^{\left(i\right)} & 0 & 0 & k_{44}^{\left(i\right)} \end{array}\right]\\ K^{\left(e\right)}=\left[\begin{array}{cccc} k_{11}^{\left(e\right)} & 0 & 0 & 0\\ 0 & k_{22}^{\left(e\right)} & 0 & 0\\ 0 & 0 & k_{33}^{\left(e\right)} & 0\\ 0 & 0 & 0 & k_{44}^{\left(e\right)} \end{array}\right]\\ k_{\mathrm{ab}}^{\left(i\right)}=\frac{1}{2}a_{\mathrm{ab}}e^{-c_{\mathrm{ab}}|x|}\\ k_{\mathrm{aa}}^{\left(e\right)}=\frac{1}{2}c_{\mathrm{aa}}\left(e^{-c_{\mathrm{aa}}\left|x-h_{a}\right|}+e^{-c_{\mathrm{aa}}\left|x+h_{a}\right|}\right) \end{array}$$

**Fig. 2:**
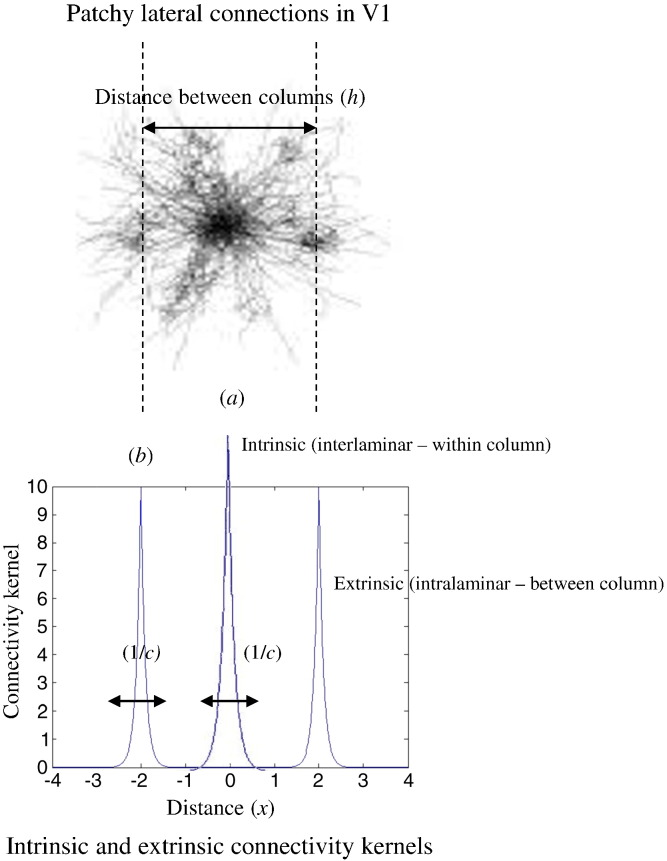
Connectivity kernel. This kernel describes a combination of patchy but isotropic distributions by using connectivity kernels with non-central peaks. It models sparse intrinsic connections in cortical circuits that mediate both local (within macrocolumn) and non-local (between macrocolumn) interactions. In other words, neurons talk both to their immediate neighbours and receive input from remote populations who share the same functional selectivity; see Eq. (8). The insert is a modified from www.ini.uzh.ch/node/23776.

**Fig. 3:**
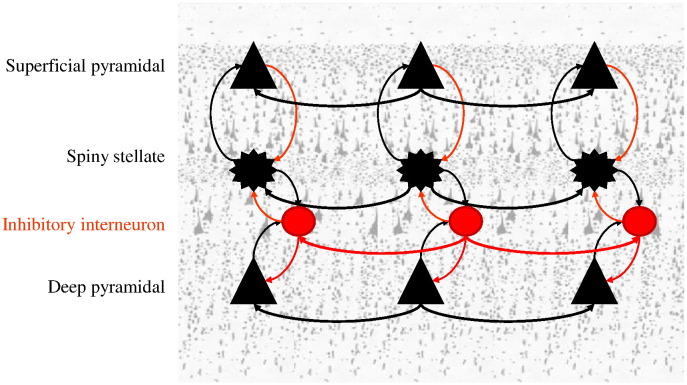
Three neighbouring macrocolumns. Each macrocolumn consists of the four subpopulations of Fig. 1 connected to each other with intralaminar (within macrocolumn) and interlaminar (between macrocolumns) connections. We later assume that the visual cortex is tiled with replications of this cortical circuitry and that individual differences in neuroanatomy are reflected in gamma frequency activity that can be attributed to a variable columnar size (the *c* parameter of Fig. 2).

Here, the parameters *a*_*ab*_ and *c*_*ab*_ encode the strength (analogous to the number of synaptic connections) and extent of intrinsic connections between cortical layers. The intrinsic connections can be regarded as interlaminar connections within a macrocolumn, while the extrinsic (between macrocolumn) connections correspond to horizontal connections and connect layers of the same type at a distance *h*_*a*_. Later, we will use the extent parameter *c*_*ab*_ as a measure of the size of cortical macrocolumns: see Fig. 2 for an illustration of the spatial parameters and Fig. 3 for a distinction between interlaminar and horizontal intra-laminar connections.

On comparing Eqs. (2) and (7) we notice that presynaptic input to the *a*-th population from the *b*-th is now expressed in terms of a spatiotemporal convolution as opposed to the simple nonlinear (sigmoid) mapping of neural mass models. For connectivity kernels modelling homogeneous (local) interactions, this convolution gives rise to wave equations describing propagation of the presynaptic input on the cortical manifold. In essence, Eq. (7) with connectivity defined by Eq. (8) defines a class of neural field models that can accommodate the intrinsic connectivity rules prescribed by the canonical microcircuit.

As in our earlier work (Pinotsis and Friston, 2011; Pinotsis et al., 2012), we assume that the neural field defined by Eq. (7) is perturbed around a spatially homogeneous steady-state *V*_0_ (attained in the absence of external or exogenous perturbations)

(9) $$V_{0}=B^{-1}A\cdot F\left(V_{0}\right)\int K\left(x\right)dx$$

Using linear systems analysis, we now define the transfer function of the field model described above by the following relation

(10) $$T\left(k,\omega \right)=\frac{P\left(k,\omega \right)}{U\left(k,\omega \right)}$$

where *U*(*k*,*ω*) is the two-dimensional Fourier transform of external input:

(11) $$\begin{array}{c} U\left(k,\omega \right)=FT\left(U\left(x,t\right)\right)\\ =\iint U\left(x,t\right)e^{-ikx+i\omega t}\text{d}t\text{d}x \end{array}$$

and *P*(*k*,*ω*) is the Fourier transform of the perturbations around the steady-state solution. Given the transfer function, we can characterise the spectral response of the system to any external input, in terms of the underlying connectivity kernel, propagation velocities and post-synaptic response function. By analogy to a single population, substituting *V*(*x*,*t*) = *V*_0_ + *P*(*x*,*t*) into Eq. (7) and expanding *F* ∘ *V* around *V*_0_, we obtain a second-order expression for the perturbations *P*(*x*,*t*)

(12) $$\begin{array}{c} \ddot{P}+2B\dot{P}=-B^{2}P+AB\cdot D\gamma ⊗P+GU\\ \gamma =\partial F/\partial V \end{array}$$

Here, *γ* is the gain of the nonlinear mapping between depolarisation and firing rate:

(13) $$\gamma_{\mathrm{ab}}=\frac{\partial \sigma \left(v_{a}=0\right)}{\partial v_{b}}=\{\begin{array}{cc} \frac{re^{\mathrm{r\eta}}}{{\left(1+e^{\mathrm{r\eta}}\right)}^{2}} & a=b\\ 0 & a\neq b \end{array}$$

Eqs. (10) and (12) provide the transfer function of our canonical microcircuit neural field model. Taking the Fourier transform of Eq. (12) and substituting into Eq. (10) gives:

(14) $$\begin{array}{c} T\left(k,\omega \right)={\left(-\omega^{2}I_{4}-2i\omega B+B^{2}-J\left(k,\omega \right)\right)}^{-1}G\\ J\left(k,\omega \right)=ABD\left(k,\omega \right)\gamma \end{array}$$

where *J*(*k*,*ω*) is a 4 × 4 matrix incorporating the synaptic parameters, connectivity parameters and gain matrix and *D*(*k*,*ω*) is the Fourier transform of the spatiotemporal connectivity. In summary, Eq. (14) provides a mathematical model or transfer function mapping from exogenous inputs or fluctuations acting upon each neuronal layer and the resulting spatiotemporal response in source space. This transfer function is specified completely by synaptic and connectivity parameters implicit in the neural field model. We next consider the mapping from source space to sensor space that completes the forward or generative model.

## A neural field DCM

### A generative model for power spectra

In what follows, we describe the generative or forward mapping from external inputs (exogenous fluctuations) to observed spectral responses. This allows one to compare the predictions of our model with real data and requires a mapping of neuronal states (the depolarisation fields above) to sensors. This mapping is called the lead field. The lead field allows one to infer hidden parameters characterising the deployment of sources on the cortical surface, even when there is no explicit spatial information in the data, see also Robinson et al. (2004). The lead field samples particular spatiotemporal frequencies, depending on the sensitivity profile of the sensors used. For example, if the lead field has a narrow spatial support (e.g., when using LFP electrodes), its spatial Fourier transform will be broad and it will be sensitive to a wide range of spatial frequencies. Conversely, when the lead field sees a large part of the cortical surface (e.g., non-invasive EEG sensors), the spatial Fourier transform will be narrow and only fluctuations in low spatial frequencies will contribute to the observed cross-spectra.

In the illustrative examples below we used an adaptive spatial filter or beamformer (Van Veen et al., 1997) to obtain estimates of ongoing neuronal activity in primary visual cortex (Schwarzkopf et al., 2012). This provides an estimate of electrical cortical activity based on a weighted combination of sensors — sometimes referred to as a virtual electrode. In other words, we will consider a single (virtual) sensor — so that the lead field maps from hidden neuronal states to a single data channel. We first describe the lead field and its parameterisation in terms of a Fourier basis set and coefficients that scale the contribution of neuronal states in each layer. We then describe how the predicted spectral response at the sensor depends upon the (Fourier transforms) of the lead field and the transfer function *T*(*k*,*ω*) above that maps external inputs to neuronal states. Given the particular form of the lead field, we can simplify the expressions for the resulting transfer function from external inputs to spectral responses. This transfer function provides the basis of our probabilistic model. In detail:The lead field is parameterised by *φ* as a continuous gain function *L*(*x*,*φ*) over the cortical patch that is applied to a mixture of neuronal (depolarisation) states at each point on the patch. This mixture is determined by four coefficients $\tilde{Q}=\left[{\tilde{q}}_{1}\;{\tilde{q}}_{2}\;{\tilde{q}}_{3}\;{\tilde{q}}_{4}\right]$, while the gain function is parameterised in terms of the coefficients *L*(*k*,*φ*) of a spatial Fourier basis set:

(15) $$L\left(x,\varphi \right)=\sum_{k}L\left(k,\varphi \right)e^{\mathrm{ikx}}$$

The predicted response at the virtual electrode — for a given set of all the neural and lead field parameters *θ* — is obtained by integrating over the cortical patch

(16) $$y\left(t,\theta \right)=\int L\left(x,\varphi \right)\left(\tilde{Q}\cdot V\left(x,t\right)\right)dx$$

which leads to a spectral response of the form

(17) $$Y\left(\omega ,\theta \right)=\sum_{k}L\left(k,\varphi \right)\left(\tilde{Q}\cdot T\left(k,\omega \right)U\left(k,\omega \right)\right)$$

The predicted spectral response measured by the sensor is therefore

(18) $$\begin{array}{c} g\left(\omega ,\theta \right)=Y\left(\omega ,\theta \right)Y^{*}\left(\omega ,\theta \right)\\ =\sum_{k}|L\left(k,\varphi \right){|}^{2}\tilde{Q}T\left(k,\omega \right)g_{u}\left(k,\omega \right)T{\left(k,\omega \right)}^{*}{\tilde{Q}}^{T} \end{array}$$

where *g*_*u*_(*k*,*ω*) = |*U*(*k*,*ω*)|^2^ is the auto-power spectrum of external input. We will consider a gain function with a simple Gaussian form, which we parameterise in terms of its dispersion *φ* such that $L\left(x,\varphi \right)=e^{-x^{2}/2\varphi^{2}}$, noting that the amplitude is fixed to avoid redundancy with the parameters $\tilde{Q}=\left[{\tilde{q}}_{1}\;{\tilde{q}}_{2}\;{\tilde{q}}_{3}\;{\tilde{q}}_{4}\right]$. This leads to Fourier coefficients of the form $L\left(k,\varphi \right)=e^{-2\pi^{2}\varphi^{2}k^{2}}$ and Eq. (19) becomes

(19) $$\begin{array}{c} g\left(\omega ,\theta \right)=\sum_{k}{\left|e^{-2\pi^{2}\varphi^{2}k^{2}}\right|}^{2}\tilde{Q}T\left(k,\omega \right)g_{u}\left(k,\omega \right)T{\left(k,\omega \right)}^{*}{\tilde{Q}}^{T}\\ =\sum_{a,k}{\tilde{q}}_{a}W_{a}\left(k,\omega \right) \end{array}$$

The second equality follows by substituting the transfer function *T*(*k*,*ω*) in Eq. (14) into Eq. (19), to express the prediction as a mixture of contributions from each population weighted by ${\tilde{q}}_{a}$:

(20) $$W_{a}\left(k,\omega \right)={\left|e^{-2\pi^{2}\varphi^{2}k^{2}}\kappa_{1}m_{e}S_{a}\left(k,\omega \right)R^{-1}\left(k,\omega \right)\right|}^{2}g_{u}\left(k,\omega \right)$$

The term *S*_*a*_(*k*,*ω*)*R*^− 1^(*k*,*ω*) in (20) expresses the relative contribution of each population to the predictions at source level and depends upon the particular form of the connections among these populations. It can be seen from Eq. (8), that this ratio depends upon the (Fourier transforms of) intrinsic and extrinsic connectivity (see also, Pinotsis and Friston, 2011; Pinotsis et al., 2012);

$$\begin{array}{c} D_{\mathrm{ab}}^{\left(i\right)}\left(k,\omega \right)=\frac{a_{\mathrm{ab}}\left(c_{\mathrm{ab}}-i\upsilon \omega \right)}{c_{\mathrm{ab}}^{2}-\upsilon_{\mathrm{ab}}^{2}\omega^{2}-2i\upsilon c_{\mathrm{ab}}\omega +k^{2}}\\ D_{\mathrm{aa}}^{\left(e\right)}\left(k,\omega \right)=\frac{c_{\mathrm{aa}}}{2}[\frac{e^{-h_{a}c_{\mathrm{aa}}}c^{-}-e^{i\upsilon h_{a}\omega -2h_{a}c_{\mathrm{aa}}}\left(c^{-}\alpha +\beta \right)}{4k^{2}\pi^{2}+c^{-}c^{-}}+\frac{e^{i\upsilon h_{a}\omega }c^{+}\alpha -e^{-h_{a}c_{\mathrm{aa}}}c^{+}+e^{i\upsilon h_{a}\omega -h_{a}c_{\mathrm{aa}}}\beta }{4k^{2}\pi^{2}+c^{+}c^{+}}]\\ \alpha =\cos\left(2h_{a}k\pi \right),\;\beta =2k\pi \sin\left(2h_{a}k\pi \right)\;\\ c^{+}=c_{\mathrm{aa}}+i\upsilon \omega ,\;c^{-}=c_{\mathrm{aa}}-i\upsilon \omega \end{array}$$

(21)

In particular, *R*(*k*,*ω*) and *S*_*a*_(*k*,*ω*) are given by

(22) $$\begin{array}{c} R\left(k,\omega \right)=-V_{14}\left(k,\omega \right)\left(-V_{23}\left(k,\omega \right)+Q_{2}\left(k,\omega \right)Q_{3}\left(k,\omega \right)\right)\\ +Q_{4}\left(k,\omega \right)\left[-V_{23}\left(k,\omega \right)Q_{1}\left(k,\omega \right)+Q_{3}\left(k,\omega \right)\left(-V_{12}\left(k,\omega \right)+Q_{1}\left(k,\omega \right)Q_{2}\left(k,\omega \right)\right)\right]\\ S_{1}\left(k,\omega \right)=-Q_{4}\left(k,\omega \right)\left(-V_{23}\left(k,\omega \right)+Q_{2}\left(k,\omega \right)Q_{3}\left(k,\omega \right)\right)\\ S_{2}\left(k,\omega \right)=D_{21}^{\left(i\right)}\left(k,\omega \right)\gamma \kappa_{2}m_{i}Q_{3}\left(k,\omega \right)Q_{4}\left(k,\omega \right)\\ S_{3}\left(k,\omega \right)=-D_{21}^{\left(i\right)}\left(k,\omega \right)D_{32}^{\left(i\right)}\left(k,\omega \right)\gamma^{2}\kappa_{2}\kappa_{3}m_{e}m_{i}Q_{4}\left(k,\omega \right)\\ S_{4}\left(k,\omega \right)=D_{41}^{\left(i\right)}\left(k,\omega \right)\gamma \kappa_{4}m_{e}\left(-V_{23}\left(k,\omega \right)+Q_{2}\left(k,\omega \right)Q_{3}\left(k,\omega \right)\right) \end{array}$$

where the functions *Q*_*a*_(*k*,*ω*) and *V*_*ab*_(*k*,*ω*) depend on the Fourier transforms *D*_*ab*_^(*i*)^(*k*,*ω*) and *D*_*aa*_^(*e*)^(*k*,*ω*) as follows:

(23) $$\begin{array}{c} Q_{a}\left(k,\omega \right)=-{\kappa_{a}}^{2}+\gamma \left(D_{\mathrm{aa}}^{\left(i\right)}\left(k,\omega \right)+D_{\mathrm{aa}}^{\left(e\right)}\left(k,\omega \right)\right)\kappa_{a}m_{a}+2i\kappa_{a}\omega +\omega^{2}\\ V_{\mathrm{ab}}\left(k,\omega \right)=D_{\mathrm{ab}}^{\left(i\right)}\left(k,\omega \right)D_{\mathrm{ba}}^{\left(i\right)}\left(k,\omega \right)\gamma^{2}\kappa_{a}\kappa_{b}m_{a}m_{b} \end{array}$$

These expressions may look complicated but can be obtained in a fairly straightforward way from Eq. (19). In summary, the predicted spectral response at the sensor is given by:

(24) $$g\left(\omega ,\theta \right)=\sum_{k}{\left|e^{-2\pi^{2}\varphi^{2}k^{2}}\kappa_{1}m_{e}\sum_{a}{\tilde{q}}_{a}S_{a}\left(k,\omega \right)R^{-1}\left(k,\omega \right)\right|}^{2}g_{u}\left(k,\omega \right)$$

Eq. (24) reflects the fact that the predicted spectral responses of the system are coupled to its *spatial* as well as its temporal properties; these properties are encoded in the transfer functions *S*_*a*_(*k*,*ω*) and *R*(*k*,*ω*) through the underlying connectivity functions *D*_*ab*_(*k*,*ω*). In turn, these are specified by the synaptic parameters associated with the canonical microcircuit *θ* ⊂ {*m*_*i*_,*m*_*e*_,*κ*_*i*_,*κ*_*e*_,*r*,*η*} and the spatial parameters *θ* ⊂ {*a*_*ab*_,*c*_*ab*_,*h*_*a*_,*υ*_*ab*_} that encode intrinsic and extrinsic connections among different layers and neighbouring columns or points on the cortical manifold.

To complete our specification of a generative model, we assume that the observed cross-spectra *g*_*y*_ are a mixture of predicted spectra, channel and Gaussian observation noise

(25) gyω=gωθ+gnωθ+εguωθ=αu+βuωgnωθ=αn+βnω,Reε~N0,ΣωλImε~N0,Σωλ

The spectra of the neuronal fluctuations or input *g*_*u*_(*ω*,*θ*) are assumed to be spatially white; namely, they do not depend on spatial frequency. However both input and noise spectra are modelled as a mixture of white and coloured fluctuations over time. In completing the model, we have introduced extra free parameters *θ* ⊂ {*α*_*n*_,*α*_*u*_,*β*_*n*_,*β*_*u*_} controlling the spectra of the inputs and channel noise. Eq. (30) provides the basis for our generative model and entails free parameters controlling the spectra of the inputs and channel noise as well as the amplitude of observation error. Gaussian assumptions about the observation error mean that we have a probabilistic mapping from all unknown (free) parameters to observed (spectral) data features. Inversion of this model means estimating, probabilistically, the free parameters given data.

### Model inversion and fitting

Having prescribed the generative model of our DCM, we can now turn to its optimisation via Bayesian techniques. Almost universally, the fitting or inversion of Dynamic Causal Models optimises variational free-energy. Variational free-energy serves as a bound approximation to the log-evidence ln *p*(*g*_*y*_|*M*) for a model *M*. This optimisation is carried out with respect to a variational density *q*(*θ*) on the unknown model parameters. By construction, the free-energy bound ensures that when the variational density maximises free-energy, it approximates the true posterior density over parameters, *q*(*θ*) ≈ *p*(*θ*|*g*_*y*_, *M*). At the same time, the free-energy approximates the log-evidence (log-marginal likelihood of the data). The (approximate) conditional density and (approximate) log-evidence are used for inference on parameters and models respectively. In other words, one first compares different models (e.g., with and without particular connections) using their log-evidence and then turns to inferences on parameters, under the model selected. One usually assumes the conditional density has a Gaussian form $q\left(\theta \right)=N\left(\mu ,C\right)$. This is known as the Laplace assumption. The conditional density is summarised by the most likely value of the parameters, *μ* and their conditional covariance *C* that encodes uncertainty about the estimates and their conditional dependencies. A full description of the resulting Variational Laplace scheme can be found in Friston et al. (2007).

The underlying generative model generally admits a unique solution during model inversion; this follows from the use of biophysically plausible priors over the biophysical parameters. Table 1 describes the priors over synaptic parameters (that are also used in the classical Jansen and Rit model) as well as parameters pertaining to the spatial structure of cortical sources. These priors are based on the modelling literature. Others come from the experimental literature; e.g. the prior for the conduction velocity is assumed to be 1.5 m/s (Kandel et al., 2000; Steriade et al., 1997). In general, priors are chosen to restrict parameter estimates in a physiologically meaningful range. However, it should be noted that the precise values of the priors are not important: the inversion scheme has the latitude to accommodate deviations from these values to optimise model evidence.

## Results — an empirical illustration and validation

In previous work (Schwarzkopf et al., 2012), we recorded visually induced gamma oscillations in a group of healthy human subjects using magnetoencephalography (MEG) and used functional magnetic resonance imaging (fMRI) to measure the surface area of central primary visual cortex (V1) with standard retinotopic mapping techniques (Dale et al., 1999; Sereno et al., 1995). We showed that the retinotopically defined surface areas of central V1 and V2 are correlated with the peak frequency of visually induced oscillations in the MEG gamma band. This led to the proposal that individual differences in macroscopic gamma frequency may be attributed to inter-individual variability in the microscopic architecture of visual cortex (Schwarzkopf et al., 2012). In this section, we use the neural field model of the previous section to address this proposal.

### Empirical data and validation

**Fig. 4:**
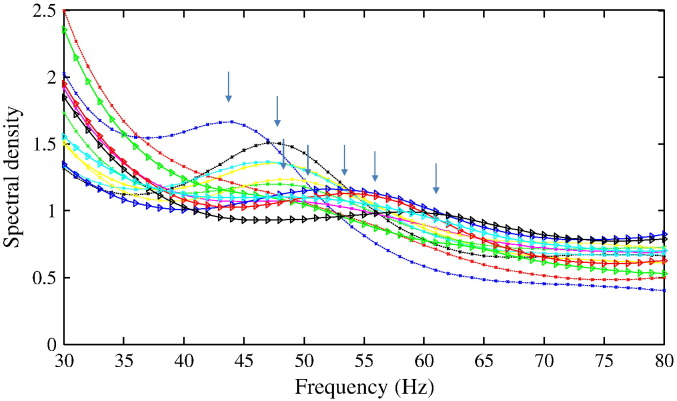
Observed MEG spectra from a selection of subjects with different V1 surface area. Schwarzkopf et al., 2012 found a strong positive correlation between gamma peak frequency (marked with arrows) and V1 surface area. We used a biophysical model to investigate whether individual differences in macroscopic gamma frequency may reflect inter-individual variability in the architecture of visual cortex.

Here, we used MEG data and beamforming to summarise the spectral expression of endogenous activity in the visual cortex of 16 subjects, see Fig. 4 and Schwarzkopf et al. (2012). Each participant was stimulated in both left and right visual fields — so we characterised separate sources in right and left hemispheres, resulting in 32 data sets. Subjects were seated in a MEG system and viewed the stimulus on a projection screen in front of them. During the entire recording run, they were required to maintain fixation on a small red dot in the centre of the screen. The stimulus was a static, high-contrast, square-wave, vertical grating, which measured 4° × 4° of visual angle and had a spatial frequency of 3 cycles per degree. The grating was presented on a mean luminance uniform gray background in the lower visual field, such that the corner closest to the centre of gaze was horizontally and vertically displaced from the fixation spot by 0.5°. There were 180 trials; on half the grating was shown in the lower right quadrant; on the other half, it was shown in the lower left. We recorded MEG data using a whole-head CTF axial gradiometer system with 275 channels, sampled at 600 Hz. Three electrical coils were placed at fiducial locations and used to monitor subject head movement. Data were analysed using SPM8 (http://www.fil.ion.ucl.ac.uk/spm). Recordings were divided into epochs from 1.5 s before stimulus onset until 1.5 s after stimulus onset (i.e., the earliest time for stimulus offset that preceded the participant's behavioural response). Employing a beamforming approach, we estimated induced MEG responses to a visual stimulus in a virtual electrode placed in the medial occipital cortex, in a location consistent with primary visual cortex. We used an LCMV beamformer algorithm (Van Veen et al., 1997) implemented in SPM8 to quantify source power in the time window between 0.5 and 1.5 s after stimulus onset relative to baseline power over one second preceding stimulus onset. Source orientation at each voxel was determined using the method of Sekihara et al. (2004). We located peak gamma activity in the medial occipital cortex, and at this peak location we used the beamformer weights to extract the time series of the virtual electrode. Power spectra for frequencies between 30 and 80 Hz during the stimulation and the baseline were calculated using a multitaper spectral estimate (Thomson, 1982) using seven discrete prolate spheroidal sequences as data tapers. To summarise the peak gamma frequency — and the amplitude and bandwidth of the gamma response — we fitted a Gaussian function to the percentage power change in each frequency bin. A full description of the paradigm, recording and processing can be found in Schwarzkopf et al. (2012).

We modelled these spectral data — using DCM — to address the following questions: (i) Are neural masses or fields more appropriate for explaining these data? (ii) Can differences in the width of cortical columns explain the intersubject differences in gamma frequency? (iii) How does cortical excitability relate to the peak gamma frequency? And (iv) what are the important determinants of spectral gamma activity? The first question illustrates the use of Bayesian model comparison to compare DCMs based on neural fields and neural masses. We will see below that — for the MEG data and generative models considered here — the evidence for neural field models is relatively weak, which itself is interesting in relation to previous results. However, our primary aim was to show how DCM can answer anatomical questions about brain topography and architecture; by appealing to neural field models that embody plausible assumptions about source deployment and microcircuitry. We illustrate this point using subject-specific estimates of intrinsic connections from DCM and establish their predictive validity in terms of their ability to predict inter-subject variations in the size of V1 and gamma activity. We consider that the visual cortex is tiled with (overlapping) macrocolumns and assume that the major influences on each macrocolumn come from its nearest neighbours. We also assume rotational symmetry so that the spatial organisation of macro columns can be modelled by the field model described in the previous section (see Figs. 2 and 3). These connections are characterised by parameters describing local synaptic arbours (like the strength *a*_*ab*_ and extent 1/*c*_*ab*_ of intralaminar connections) and the range of horizontal connections (*h*_*a*_). Our objective was to provide a mechanistic link between observed cross-spectral densities and local microstructure: we used the spatial decay rate of horizontal interlaminar connections *c*_*ab*_ as an estimate of column width.

### Neural masses or fields?

Clearly, the choice of an appropriate model depends upon the question of interest; in particular, neural fields are appropriate for addressing questions about the deployment of sources on the cortical surface and induced spatial dynamics. However, neural field models might still be more appropriate from a Bayesian perspective, even if the spatial parameters of a neuronal model are not the focus of study: In the context of our Bayesian scheme, each model is scored using a free energy bound on model-evidence, where better models have a higher free energy (assuming free energy is a good approximation to model evidence). The model with the highest evidence implies the model has an optimal balance between accuracy and complexity — in the sense that the model provides an accurate explanation for the data in the simplest way. In our earlier work, we showed that Bayesian model selection can distinguish between neural mass and field variants of the same microcircuitry; where — for LFP data from the rat auditory cortex — the neural field variant had more evidence. This could be explained by the fact that neural field models provide an augmented repertoire of predictions for dynamics resulting from the propagation of activity on the cortical surface. In short, our earlier model comparisons showed that the neural field model yields a better fit to the LFP data — while accounting for the complexity cost due to its extra free parameters.

**Fig. 5:**
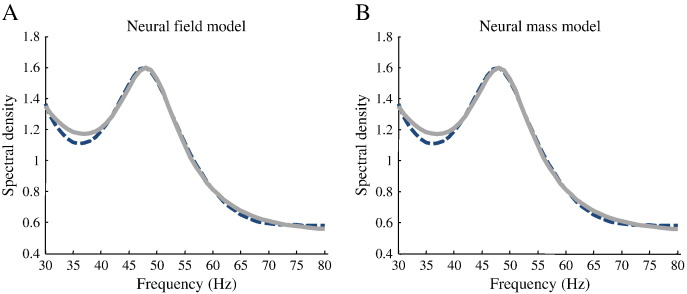
Example of DCM fits for a single participant. Real data (dashed line) and model predictions (full line) for spectra in the gamma band obtained from the human visual cortex during visual stimulation (Schwarzkopf et al., 2012). We observe that the fits of both the field and mass models are equally good with no manifest differences.

**Fig. 6:**
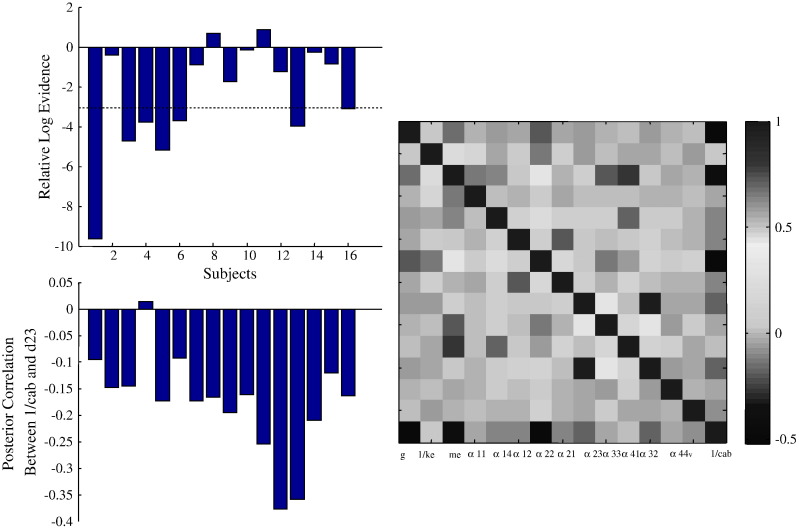
(Top panel) Bar chart of relative log evidence for neural mass and field models over subjects. The average relative log evidence was 2.37 in favour of the mass models, which is considered insufficient for disambiguating between models: only a relative evidence of three (dashed line) constitutes strong evidence a particular model offers a better explanation for the data. Note that in 6 out of 16 individuals evidence in favour of the mass model was greater than three. (Bottom panel) Left: Bar chart of posterior cross-correlations between columnar width and the connection strength between deep pyramidal cells and inhibitory interneurons. The average cross-correlation was − 0.176. Right: Mean over subjects of the posterior cross–correlation matrices for all parameters in our neural field model.

Here, we performed a similar analysis, by computing the relative log-evidence (using the free energy approximation) for the canonical microcircuit model, comparing field and mass variants at the group level. Synaptic (*κ*_1_, *m*_*e*_, *a*_*ab*_) spatial (*υ*, *c*_*ab*_) and sigmoid (*r*, *η*) parameters were optimised, while the remaining parameters in Table 1 including the horizontal distance *h*_*a*_ were fixed to physiologically plausible values. As with our previous model comparison, the neural mass model was formulated as a special (and limiting) case of the neural field model by shrinking the propagation delays (conduction times) times to zero. See Fig. 5 for an example of a model fit to a single subject's response, using the neural field and mass variants. Contrary to our earlier result, we found inconsistent evidence in favour of the neural mass model (the log-evidence for the neural mass model was on average 2.37 greater than that of the field model): see Fig. 6 (top panel). In relation to our previous model comparisons, this result highlights the fact that the best model depends upon the data analysed. It also underlines the importance of combining a neuronal model with a spatial forward model: although both auditory and visual cortices are thought to conform to the local homogeneity constraints implicit in neural field models, the loss of spatial frequency resolution — with non-invasive data — might render neural field models unnecessary, in relation to neural mass models. In brief, model evidence depends crucially upon both the chosen models and the particular dataset used for their comparison. Our failure to establish a greater evidence for neural field models, in the present model comparison, is intuitively sensible because non-invasive MEG data have much lower spatial resolution than the LFP data we used in the previous model comparison. This observation speaks to the potential importance of using spatially resolved data to take full advantage of neural field models: data with high signal to noise ratio and wide brain coverage — such as those afforded by ECoG sensors or multi-array grids — can, in principle, disclose a full spectrum of spatiotemporal dynamics at different scales. This may be important for an informed (efficient) estimate of spatial parameters in neural field models. We will illustrate this point further in future work.

To assess conditional dependencies among parameter estimates, we computed the mean of posterior correlation matrices across all subjects, see Fig. 6 (right) and considered a correlation between a particular pair of parameters (one spatial and one neuronal — bottom left): the key thing to notice here are the relatively weak correlations between posterior estimates of columnar width and *a*_23_ (the strength of connections from deep pyramidal cells to inhibitory interneurons). We will focus on this connection later when assessing the importance of spatial and synaptic parameters in predicting peak gamma frequency.

Finally, we characterised the variations in spectral profile produced by changes in these two parameters about their posterior estimates. Our aim here was not to provide a systematic characterisation of the neural field model; for this we refer the reader to earlier work (Nunez, 1995; Robinson et al., 2001, 2003, 2004; Valdes et al., 1999). We just wanted to provide a demonstration of the complicated but smooth contribution to spectral responses made by various parameters. It is this contribution that enables us to obtain informed estimates of underlying microcircuitry. Note that increasing both the spatial and neuronal parameters increases peak gamma frequency. In other words, increasing the spatial extent of local horizontal connections and increasing the excitatory drive to inhibitory interneurons increases peak gamma frequency. However, these parameters show (weak) negative conditional correlations. This means it is an open question as to which parameter is best able to account for changes in peak gamma frequency over subjects.

### Can individual differences in peak gamma frequency be attributed to wider columns?

Our conventional analyses (Schwarzkopf et al., 2012), showed a strong positive correlation between gamma peak frequency and V1 surface area. This implies that individual differences in V1 area might reflect differences in cortical architecture. A larger cortex could either comprise a similar number of wider columns or simply contain a greater number of columns of constant width, which would be consistent with the explanation offered by the model of Prothero, (1997). Anatomical evidence suggests that the micro-architecture of V1 in humans resembles a scaled version of macaque V1, implying that column width increases with V1 size across species (Adams et al., 2007). However, across individuals of the same species both the width and number of columns can be highly variable (Horton and Hocking, 1996). Crucially, we can resolve this issue directly given the parameterisation of the intrinsic connectivity within the neural field model. In our model, the width of a macrocolumn, corresponds to the dispersion of horizontal synaptic connections 1/*c*_*ab*_.

**Fig. 7:**
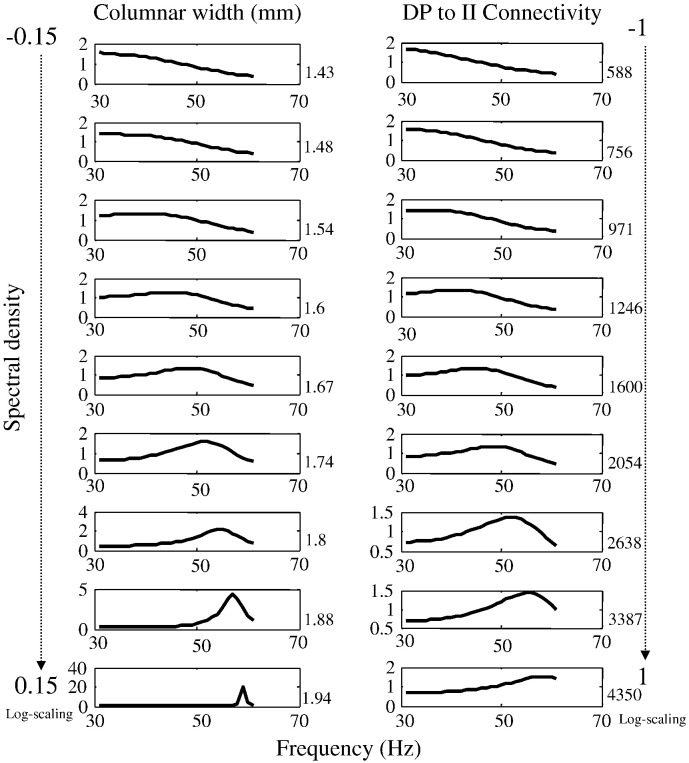
Contribution analysis of particular parameters. This figure shows changes in spectral responses elicited by varying the lateral extent of intrinsic connectivity and the strength of intrinsic connections between interneurons and deep pyramidal cells (over a log-scaling range).

**Fig. 8:**
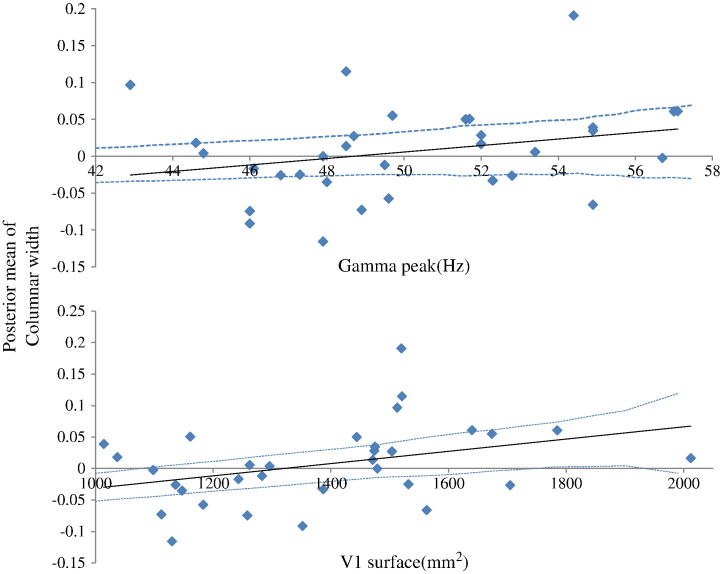
(Top panel): We found a correlation between the log scaling of the posterior estimate of columnar width and peak gamma frequency (Pearson *r* = 0.271, *p* = 0.06, 30 d.f., one-tailed test). This suggests that increases in peak gamma frequency across subjects can be attributed to a greater columnar width. (Bottom panel) Correlation between the log scaling of the posterior estimate of columnar width and V1 surface (Pearson *r* = 0.36, *p* = 0.02, 30 d.f., one-tailed test). This result suggests that a larger V1 is constituted by bigger macrocolumns. This finding, together with that reported in the top panel, confirm our earlier empirical finding that gamma peak and V1 size are correlated. Furthermore, our use of an underlying generative (mechanistic) model, offers insight into local cortical microstructure that would be hard (or impossible) to disclose otherwise. Confidence intervals are plotted as dotted lines.

We found a correlation between columnar width and peak gamma frequency that reached trend significance (see top panel of Fig. 8). Given our previous empirical finding (that peak gamma frequency and V1 surface area are positively correlated) we considered a one-tailed test and found Pearson *r* = 0.271, *p* = 0.06, (d.f. 30). This correlation implies that variability in the observed spectral responses in the gamma range can be attributed to individual differences in columnar width. This finding is more than simply noting that increasing the size of a column increases peak gamma frequency under the neural field model (see Fig. 7). This is because all the other free parameters of the model were optimised in a subject-specific fashion. In short, the hypothesis that intersubject variations in columnar width are associated with peak gamma frequencies was confirmed thereby providing a microscopic explanation for the correlation between peak gamma frequency and macroscopic (retinotopic) measurements of visual cortex anatomy — an explanation based purely on non-invasive MEG data.

We also found a significant positive correlation between columnar width and V1 size as measured with retinotopic mapping (see bottom panel of Fig. 8, where Pearson *r* = 0.36, *p* = 0.02, 30 d.f., — one-tailed test). This suggests that subjects with a larger V1 have wider as opposed to more macrocolumns. The results in Fig. 8 are consistent with our previous empirical finding of a significant correlation between observed gamma peak and size of the visual cortex (Schwarzkopf et al., 2012); however, the mediation of this correlation can now be associated with the organisation underlying cortical circuitry. This highlights the use of a generative (mechanistic model) to characterise cortical microstructure that would otherwise be hard or impossible to disclose.

### Is gamma activity related to GABAergic connections?

**Fig. 9:**
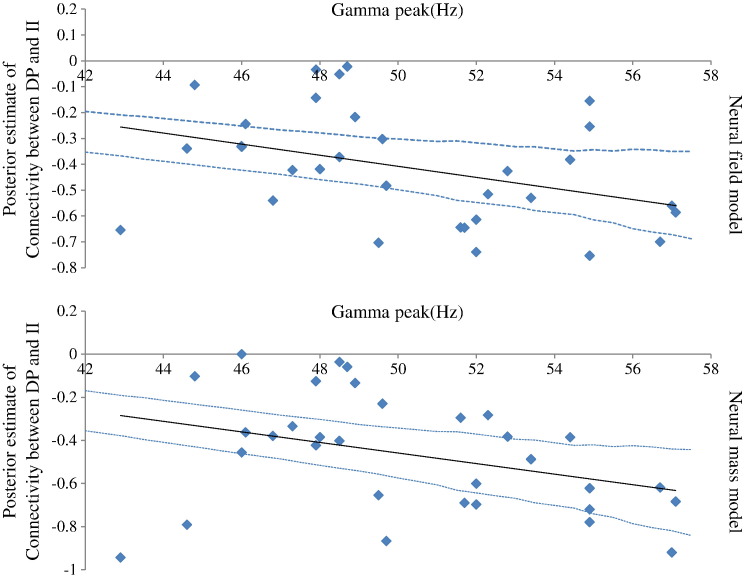
Correlation between the log scaling of the connectivity estimate between pyramidal cells and inhibitory interneurons for the neural field (Pearson *r* = − 0.37, *p* = 0.02, 30 d.f., two-tailed test) and the neural mass model (Pearson *r* = − 0.36, *p* = 0.04, 30 d.f., two-tailed test). Confidence intervals are plotted as dotted lines.

We next considered the relation of individual differences in GABA concentration and peak gamma frequency (Gaetz et al., 2011; Muthukumaraswamy et al., 2009,2010) in terms of parameters describing synaptic transmission: Muthukumaraswamy and colleagues showed that individual differences in gamma oscillation frequency are positively correlated with resting GABA concentration in visual cortex — as measured with magnetic resonance spectroscopy. Furthermore, they showed that fMRI responses are inversely correlated with resting GABA and that gamma oscillation frequency is strongly inversely correlated with the magnitude of the BOLD response. These results were taken to suggest that the excitation/inhibition balance in visual cortex is reflected in peak gamma frequencies at rest. We address this hypothesis by looking for correlations involving the connections to inhibitory interneurons. We found that posterior estimates of the excitatory connections between deep pyramidal cells and inhibitory interneurons correlated negatively with gamma peak using both the neural field model (Pearson *r* = − 0.37, *p* = 0.02, 30 d.f, two-tailed test) and the neural mass model (Pearson *r* = − 0.36, *p* = 0.04, 30 d.f, two-tailed test), see Fig. 9. This confirms the hypothesis that inter-subject variation in inhibitory drive in visual cortex is associated with characteristic changes in the peak frequency of gamma oscillations — and provides a mechanistic link from a synaptic level description to spectral behaviour that can be measured noninvasively. In summary, significant correlations were found between *a*_23_ and peak gamma frequency and columnar width and V1 size. Also, trend significance was observed for gamma peak to width correlations.

### What are the important determinants of gamma peak frequency?

Previous studies suggested two possible causes for the inter-subject variability in gamma peak frequency. Muthukumaraswamy et al. (2009) suggested that peak gamma frequency is determined by the level of inhibition in V1 as described by resting GABA concentration measured with MR spectroscopy. In our previous study (Schwarzkopf et al., 2012), we found a correlation between V1 size and peak gamma frequency and suggested that the size of V1 and associated differences in microanatomy could be true determinants of peak gamma frequency. This suggests that both GABA concentration and V1 size can influence gamma frequency; however they these factors may or may not be causally linked.

Biophysical parameters estimated using DCM provide an opportunity to investigate alternative explanations of phenotypic differences, like gamma peak frequency: our contribution analysis above (Fig. 7) shows that the excitatory drive to inhibitory neurons (*a*_23_) and macrocolumn width could mediate differences in peak gamma frequency. We therefore looked at the correlations over subjects between peak gamma frequency (f), V1 surface area and the posterior estimates of these parameters. These correlations are summarised in Table 2:

Interestingly, the partial correlation between *a*_23_ and gamma peak remained significant when controlling for V1 size and width 1/*c*_*ab*_ (*r* = − 0.332, *p* = 0.037). This suggests that the correlation between gamma peak and V1 inhibition cannot be accounted for completely by the spatial parameters (at the microscopic or macroscopic level). To elucidate the relationship between key model parameters and the phenotypes of V1 size and peak gamma frequency, we used Structural Equation Modelling (SEM) as implemented in SPSS Amos software (IBM). Each candidate model corresponded to a particular hypothesis about the causal relationships between peak gamma frequency and other variables. The Akaike Information Criterion (AIC) was used to compare models, while accounting for differences in model complexity.

**Fig. 10:**
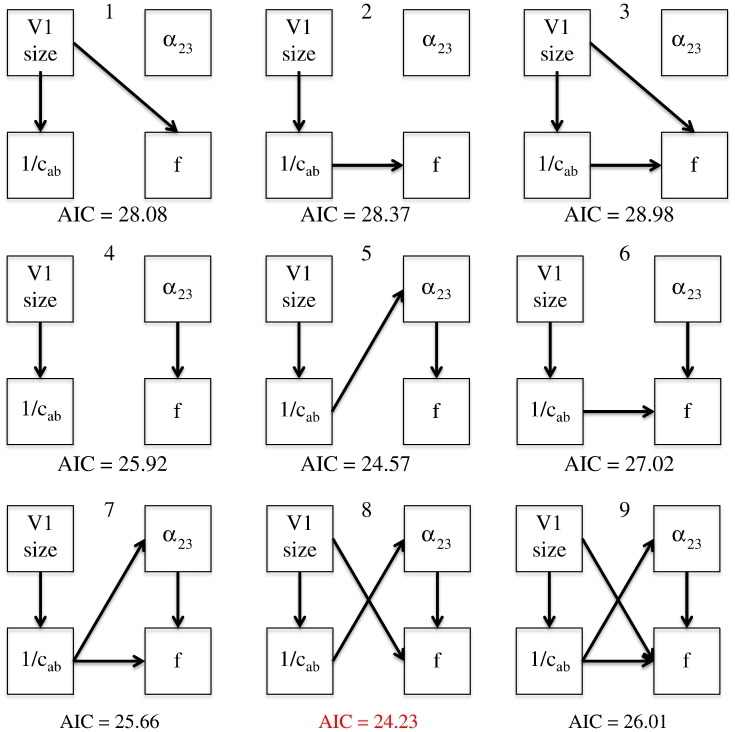
Model space for structural equation modelling. In the winning model — model 8 — the correlation between V1 size and peak gamma frequency is mediated by three links involving posterior estimates of columnar width and connection strength.

Fig. 10 shows models tested and the associated AIC values. To simplify the model space, we assumed that V1 size is determined genetically (or epigenetically) and is not influenced by peak gamma frequency or microscopic parameters. Furthermore, we assumed that the peak gamma frequency is determined by the microscopic or macroscopic anatomy. Although one could argue that these are assumptions are too simplistic — given the circular causality implied by experienced dependent plasticity — they are sufficient for our illustrative purposes. The key question here is whether peak gamma frequency is determined by (macroscopic on microscopic) spatial parameters, connectivity parameters or both.

It can be seen immediately from Fig. 10 that models without the *a*_23_→ *f* link (the top row) all have lower evidence (higher AIC) than models in which peak gamma frequency depends upon the drive to inhibitory cells. This is consistent with the significant partial correlations we found between *a*_23_ and *f* above. The best model (Model 8) captured the correlation structure between the variables very well, as can be seen from Table 3 showing the predicted and observed correlations (in parentheses).

In this model, the correlation between V1 size and peak gamma frequency is mediated by a direct path (reflecting the influences of V1 size on peak gamma frequency that can be attributed to variables not considered in these models) and an indirect path through columnar width and excitatory connections. In other words, peak gamma frequency is mediated proximately by excitatory drive to inhibitory (GABAergic) interneurons and the strength of this drive is determined, in part, by the size of macrocolumns. In turn, the size of the macro columns is constrained by the macroscopic (retinotopic) size of V1. This structural equation modelling suggests a causal link between wider macrocolumns and an increase in inhibitory drive — a hypothesis which we will come back to in the discussion.

## Discussion and conclusions

By exploiting a combination of neural field modelling and Bayesian inference, we have shown that dynamic causal modelling can answer the following sorts of questions: which is the best biophysical model for explaining electrophysiological data? What are the important determinants of gamma peak frequency — in terms of synaptic parameters and horizontal interactions? Can MEG beamformed data help us access local cortical microstructure? And how can we distinguish between competing hypotheses about structure-function relationships? We have focused on two classes of biophysical models of brain activity; so-called neural field and mass models and have considered their application to modelling empirical MEG data. Bayesian model comparison — using a variational free energy approximation to model evidence — suggests neural field models provide a better explanation of empirical data if, and only if, there is sufficient spatial frequency information in the data. In other words, we found greater evidence for neural field models in previous analyses of LFP data but failed to find more evidence for neural field models, relative to neural mass models, in the current study of MEG (virtual electrode) data. The key distinction between these different modalities is that the LFP data is sensitive to a wide range of spatial frequencies and the temporal fluctuations that these frequencies contain. In contrast, the lead fields inherent in non-invasive electromagnetic recordings are necessarily broader and suppress temporal dynamics that are expressed in high spatial frequencies.

However, the use of neural field models may be necessary when testing hypotheses that are framed in terms of the spatial parameters of neural fields — like lateral or horizontal connections: we illustrated this using a neural field DCM to explain inter-subject variations in spectral activity in terms of synaptic and connectivity parameters. We showed that the posterior estimates of cortical anatomy — in particular columnar width — are associated with the spectral peak in the gamma range. Indeed, the estimates of column width made from a single time-series estimate in the foveal portion of V1 correlates directly with size of V1 as estimate from retinotopic mapping. This constitutes a compelling illustration of how non-invasive data can provide quantitative estimates of the spatial properties of neural sources and explain systematic variations in the dynamics those sources generate. We also found correlations, over subjects, between the peak gamma frequency and cortical inhibition as parameterised by the excitatory drive to inhibitory cell populations. This correlation was motivated by previous studies looking at the three way relationship between resting GABA concentrations in visual cortex, characteristic gamma activity and excitability — as measured with evoked responses using fMRI (Muthukumaraswamy et al., 2009).

### Implications for visual perception

While visually induced gamma oscillations have received increasing attention and appear to play a role in visual perception (Herrmann et al., 2010), it remains unclear what determines the spectral properties of an individual's gamma response, and how it relates to underlying visual cortex microcircuitry. Characterising individual differences in cortical micro-architecture may have important implications for research on visual processing. A few studies have claimed to directly visualise columnar architecture in human visual cortex (Goodyear and Menon, 2001; Goodyear et al., 2002; Yacoub et al., 2008). These studies rely on ultra-high field fMRI and are technically challenging and therefore not available for widespread use. Furthermore, while the spatial resolution of these experiments may be sufficient to identify cortical columns it may be unable to reliably resolve individual differences in the column width. This limits such investigations to invasive optical imaging of intrinsic signals or voltage-sensitive dyes in animal models; yet typically animal studies do not focus on individual differences across large groups. A reliable biomarker of columnar architecture — readily measurable with non-invasive techniques — may therefore be very useful for investigating individual differences in cortical micro-architecture in human subjects.

Previous research shows that macroscopic measures of V1 size predict visual perceptual ability such as Vernier discrimination (Duncan and Boynton, 2003). We recently showed that the size of V1 is negatively correlated with the strength of visual illusions, where the conscious perception of a visual stimulus is modified by the spatial context in which it is presented (Schwarzkopf et al., 2011a, 2011b). We interpreted these results as evidence that local contextual interactions in V1 are weaker in individuals with a large V1 area — because they have to be transmitted laterally over greater distances. Furthermore, the size of population receptive fields (pRF) — which reflect a combination of the size and positional scatter of the receptive fields of single neurons — as well as the extent of contextual interactions beyond the classical receptive field — are smaller in subjects with a large V1 (Harvey and Dumoulin, 2011). This implies a greater tendency to favour local processing in a large V1. Consistent with this, orientation discrimination is also superior in individuals with a large V1 (Schwarzkopf et al., 2011a). Our results imply that better orientation discrimination could be explained by both wider cortical columns and stronger lateral inhibition. This hypothesis is also supported by the empirical work of (Edden et al., 2009) who found that GABA concentration correlates with both gamma peak frequency and orientation discrimination ability; we will focus on this issue in future work.

In summary, individuals with larger retinotopic visual cortices might have weaker contextual interactions and more extended spatial processing. Both these findings are consistent with a greater dispersion of horizontal connections, inferred on the basis of our DCM results. Interestingly, gamma oscillation frequency is also correlated with orientation discrimination ability, which suggests that orientation discrimination, V1 size, pRF size, GABA concentration and gamma oscillations are all inter-related. We hope to have shown how Dynamic Causal Modelling with neural fields may provide a quantitative and mechanistic approach to such correlations among cortical structure and function.

## Figures and Tables

**Table 1:** Prior expectations of model parameters (The spatial parameters assume the cortical patch has a diameter of 25 mm).

Parameter	Physiological interpretation	Prior mean
*m*_*e*_, *m*_*i*_	Maximum postsynaptic depolarisation	8, 32 (mV) ^a^
*κ*_1_, *κ*_2_, *κ*_3_, *κ*_4_	Postsynaptic rate constants	1/2, 1/2, 1/16, 1/28 (ms^− 1^) ^a^
*a*_22_, *a*_33_, *a*_41_ *a*_12_, *a*_44_, *a*_23_, *a*_32_ *a*_11_, *a*_14_, *a*_21_	Amplitude of intrinsic connectivity kernels	3200 800,800,1600,1600 9600,4000,4800
*c*_*ab*_	Spatial decay of connectivity kernels	$\left\{ \begin{array}{cc} 0.6 & \text{a}\neq b\\ 2 & a=b \end{array}\right.$ (mm^− 1^) ^b^
*h*_*a*_	Separation between columns	4.5 (mm)^b^
*r*, *η*	Parameters of the postsynaptic firing rate function	0.54, 0^a^
*υ*	Inverse conduction speed	0.6 s/m^b^
*ϕ*	Dispersion of the lead field	$\sqrt{2}/20$
${\tilde{q}}_{1},{\tilde{q}}_{2},{\tilde{q}}_{3},{\tilde{q}}_{4}$	Relative layer contributions	10, 0,10,80

a Wendling et al., 2000.

b Kandel et al., 2000.

**Table 2:** Correlations between key model parameters, V1 size and peak gamma frequency (*N* = 32).

	Width	*V*1 *size*	*a*_23_	*f*
Width Pearson correlation Significance	1			
*V*1 *size* Pearson correlation Significance	.364 .02	1		
*a*_23_ Pearson correlation Significance	− .32 .037	− .099 .295	1	
*f* Pearson correlation Significance	.271 .06	.286 .056	− .379 .016	1

**Table 3:** Predicted and observed correlations (in parentheses) obtained by Structural Equation Modelling.

	*V*1 *size*	Width	*a*_23_	*f*
*V1 size*				
Width	.36 (.36)			
*a*_23_	− .12 (− .1)	− .32 (− .32)		
*f*	.29 (.29)	.2 (.27)	− .38 (− .38)	
